# Baicalin—Current Trends in Detection Methods and Health-Promoting Properties

**DOI:** 10.3390/ph16040570

**Published:** 2023-04-10

**Authors:** Agata Bajek-Bil, Marcelina Chmiel, Aleksandra Włoch, Monika Stompor-Gorący

**Affiliations:** 1 Faculty of Chemistry, Rzeszow University of Technology, 35-959 Rzeszów, Poland; 2 Institute of Medical Sciences, University of Rzeszów, 35-959 Rzeszów, Poland; 3 Department of Physics and Biophysics, Wrocław University of Environmental and Life Sciences, 50-375 Wrocław, Poland

**Keywords:** baicalin, detection methods, liquid chromatography, biosensors, pharmacological effects, *Scutellaria baicalensis*

## Abstract

Baicalin (7-D-glucuronic acid-5,6-dihydroxyflavone) belongs to natural flavonoids extracted from the roots of *Scutellaria baicalensis*, the plant used in traditional Chinese medicine. It has been proven that baicalin has various pharmacological activities, such as antioxidant, anti-inflammatory, anticancer, antibacterial, and anti-apoptotic ones. However, it is essential not only to determine the medical usefulness of baicalin, but also to find and develop the most effective methods for its extraction and detection. Therefore, the aim of this review was to summarize the current methods of detection and identification of baicalin and to present the medical applications of baicalin and the underlying mechanisms of its action. Based on the review of the latest literature, it can be concluded that liquid chromatography alone or together with mass spectrometry is the most commonly used method for the determination of baicalin. Recently, also new electrochemical methods have been established, e.g., biosensors with fluorescence, which have better detection limits, sensitivity, and selectivity.

## 1. Introduction

**Figure 1:**
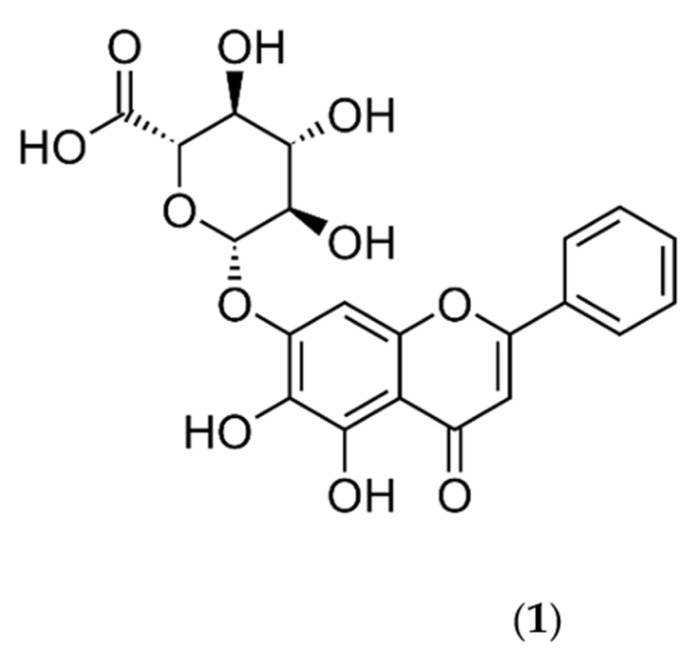
The chemical structure of baicalin (**1**).

Baicalin (**1**) (Figure 1) is the most abundant flavonoid component of *Scutellaria baicalensis* (SB) and its dried roots, known as Huang-Qin or Scutellariae Radix (SR).

It is also widely found in many multi-herbal formulations used in Eastern countries [1]. Its content in raw *Scutellaria baicalensis* Georgi roots determined by HPLC was found to be either 10.11% or 10.63%, depending on the extraction method [2].

### 1.1. Detection Methods

Many traditional Chinese medicines (TCM) are composed of SB and other herbs. Therefore many quantitative methods were developed recently for simultaneously determining their active constituents, including baicalin. The detection methods comprise ultra-high-performance liquid chromatography (UHPLC), high-performance liquid chromatography (HPLC) in combination with mass spectrometry (MS), diode array detectors (DAD), photodiode-array detectors (PDA), and also chemometric methods for the analysis of herbal formulas [3,4,5,6,7,8,9] (Table 1). In addition, liquid chromatography–tandem mass spectrometry (LC-MS/MS) was used for rat plasma analysis [10,11,12]. Similar methods are applied to determine the content of flavonoids and other ingredients of SB and its dried root SR [13,14,15,16,17,18]. A simple and accurate LC-MS/MS method was also described for the fingerprint analysis and identification of SR in many TCMs [19]. A variety of separation techniques that are employed for the quantitative determination of four main active components of SB: baicalin, baicalein, wogonin, and oroxylin A, in various types of samples were reviewed by Li et al. [18]. These techniques include HPLC, high-speed countercurrent chromatography (HSCCC), thin layer chromatography (TLC), capillary electrophoresis (CE), and micellar electrokinetic capillary chromatography (MEKC).

#### 1.1.1. Chromatographic Methods of Detection

A sensitive and selective method for the simultaneous determination of wogonin, scutellarin, baicalin, and baicalein in the SB commercial extracts using HPLC-MS/MS with electrospray ionization was developed [15]. The analysis was performed using reversed-phase chromatography with an Acclaim RSLC C18 adsorbent and gradient mixture of 0.5% aqueous formic acid solution and acetonitrile as the mobile phase. For baicalin, a 1 ng/mL limit of detection (LOD) was achieved, and the calibration curve was linear within the concentration range of 20–500 ng/mL. A UHPLC-PDA method has been developed to simultaneously determine ten flavonoids, including baicalin, in crude and wine-processed SR, which can be used as a valid analytical method for intrinsic quality control of these two preparations [16]. Optimal separation was achieved using gradient elution with the mobile phase consisting of 0.01% aqueous formic acid and methanol and a Waters ACQUITY UHPLC BEH C18 column. The detection wavelength was set at 275 nm. The measured LOD and limit of quantification (LOQ) for baicalin were 0.16 μg/mL and 0.48 μg/mL, respectively. In 2020 baicalin and sixteen other components were identified as discriminatory chemicals between raw and wine-processed SB using ultra-performance liquid chromatography/quadrupole time-of-flight mass spectrometry (UPLC-Q-TOF-MS) coupled with multiple statistical strategies [13]. A rapid, sensitive, and selective UPLC-ESI-MS/MS method was developed for the simultaneous determination of 10 flavonoids: scutellarin, scutellarein, chrysin, wogonin, baicalein, apigenin, wogonoside, oroxylin A-7-O-glucuronide, oroxylin A and baicalin from RS aqueous extracts in rat plasma, with propylparaben as an internal standard (IS). Chromatographic separation was achieved on a C18 column using gradient elution with the mobile phase consisting of methanol and 0.1% aqueous formic acid. This method was used for the pharmacokinetic comparison of crude and wine-processed RS aqueous extracts [17]. In the same year, a novel free radical reaction combined with HPLC–PDA–ESI–MS/MS screening method for the detection and identification of natural antioxidants from whole SB was described [20]. Six compounds, including baicalin and whole Scutellariae extracts, were found to possess a high potential antioxidant capacity.

The baicalein, baicalin, wogonin, and wogonoside in SR were also determined also using the reversed-phase liquid chromatographic method with isocratic elution [21]. Chromatographic analysis was performed on a YMC Pack Pro C8 column (150 × 4.6 mm, 3 mm), with a mobile phase of 0.1% aqueous formic acid and acetonitrile and UV detection at 280 nm. In 2020 Zhu et al. [22] applied multi-dimensional and multi-informational (MD-MI) integrated xanthine oxidase and superoxide anion fingerprint inequality evaluation of SR. This system, combined with HPLC-ESI-Q-TOF-MS analysis, can identify 17 active compounds in SR. In the same year, Zhang et al. [14] introduced the UHPLC-Q-TOF-MS method coupled with an integrated strategy involving diagnostic ions, neutral losses, and a prediction platform to explore the constituents of SR and their exogenous substances in rats.

All the above-mentioned methods and systems were used to examine multi-component mixtures containing baicalin.

However, over the past decade, a few of the described methods or new evaluation methods for the detection of baicalin alone or together with one to three other components were published. Among these methods, liquid chromatography is the most commonly used for the determination of baicalin in biological samples, including rat, rabbit, or human plasma. A simple, specific, and accurate method of detecting baicalin in SB Georgi using HPLC with a Diamonsil C18 column and the mobile phase consisting of methanol, water, and phosphoric acid (47:53:0.2) and the detection wavelength of 280 nm was established [23]. The content of baicalin showed linearity over the range of 0.12–1.2 μg, with an average recovery of 98.6% and a relative standard deviation (RSD) of 0.78%. Some chromatographic methods have been applied to determine and monitor baicalin content in biological samples for the purpose of pharmacokinetics. A novel UPLC-DAD method for simultaneous determination of three flavonoid glycosides: baicalin, oroxylin A-7-O-glucuronide, and wogonoside in rat plasma, using rutin as an internal standard, was published in 2015 [24]. Separation was performed on an Agilent Eclipse Plus C18 column (2.1 × 50 mm, 1.8 μm), using gradient acetonitrile and 0.2% aqueous formic acid solution as a mobile phase and with detection at 275 nm. The method was linear over the range of 0.075–17.50 μg/mL for baicalin. Its LOD in rat plasma was 0.01 μg/mL, whereas the LOQ was 0.035 μg/mL. This newly developed and validated plasma assay method has been successfully applied to the pharmacokinetic studies of baicalin, oroxylin A-7-O-glucuronide, and wogonoside after oral administration of Yinhuang granule, and the determination of baicalin in rat plasma, following oral administration of pure baicalin and SR. Wei et al. [25] described a simple and sensitive reverse-phase LC-UV analytical method to investigate the pharmacokinetics and biodistribution pattern of baicalin in rabbit plasma and tissue. The assay method is also suitable for the quantitative determination of baicalin in biosamples in preclinical and clinical experimental phase studies of baicalin-loaded liposomes. Chromatographic separation was achieved on a reverse-phase C18 column with a gradient elution with the mobile phase consisting of a 1: 1 (*v*/*v*) mixture of methanol and acetonitrile and 0.4% (*v*/*v*) aqueous phosphoric acid. UV absorption was set at 278 nm. The chromatographic response was linear over the ranges of 0.05–10.00 μg/mL in plasma and 0.05–300.00 μg/g in tissues with the LOQ of 50.0 ng/mL in plasma and tissues, and the LOD of baicalin in biosamples of 15 ng/mL. Baicalein and its main metabolite, baicalin, were also simultaneously determined in human plasma by HPLC-MS/MS method [26]. The mobile phase consisted of an aqueous phase (0.5% formic acid in 3 mM ammonium acetate solution), an organic phase (methanol-acetonitrile-formic acid, 50: 50: 0.5, *v*:*v*:*v*), and a Phenomenex^®^ SynergiTM MAX-RP 80 Å C12 chromatographic column (150 mm × 2.0 mm, 4 μm particle size) was chosen. The desired sensitivity with LOQ of 1 ng/mL was achieved, showing superior sensitivity in comparison with the methods reported previously. The method was successfully applied in the study exploring the food effect in the pharmacokinetics of baicalein chewable tablets in healthy volunteers. Intake of food before administration of baicalein chewable tablets increased the absorption of baicalein and decreased the absorption of baicalin. A sensitive LC-MS method for direct analysis of flavonoid glucuronides: baicalin, wogonoside, and apigenin-7-O-glucuronide in the bile and blood samples was described recently [27]. The analytes were separated on a Resteck HPLC (50 mm × 2.1 mm ID, 1.7 μm) column using acetonitrile and 0.1% formic acid in water as the mobile phases. The mass analysis was performed in an AB Sciex 5500 Qtrap mass spectrometer via multiple reaction monitoring (MRM) in the positive mode. The linear ranges of analytes were 10–5000 nM in the bile and 1.56–4000 nM in the blood, respectively. The validated method was successfully applied to a portal vein infusion study in rats to quantify baicalin, wogonoside, and apigenin-glucuronide in the bile and blood samples.

It is worthy of mention that baicalin determination by attenuated-total-reflectance mid-infrared (ATR-IR) and near-infrared (NIR) spectroscopy [28] in SR was also reported.

**Table 1:** Detection methods of baicalin (**1**).

Detection Methods		
Ultra-high-performance liquid chromatography (UHPLC)		
		Refs.
Ultra-high-performance liquid chromatography with photodiode-array detectors	UHPLC-PDA	[16]
Ultra-performance liquid chromatography/quadrupole time-of-flight mass spectrometry	UPLC-Q-TOF-MS	[13]
Ultra-performance liquid chromatography–electrospray ionization with mass spectrometry	UPLC-ESI-MS/MS	[17]
Ultra-high-performance liquid chromatography /quadrupole time-of-flight mass spectrometry	UHPLC-Q-TOF-MS	[14]
Ultra-performance liquid chromatography with diode array detectors	UPLC-DAD	[5,24]
Ultra-performance liquid chromatography with mass spectrometry	UPLC-MS	[27]
High-performance liquid chromatography (HPLC)		
High-performance liquid chromatography with mass spectrometry	HPLC	[23]
High-performance liquid chromatography with mass spectrometry	HPLC-MS/MS	[15,26]
High-performance liquid chromatography photodiode-array with electrospray ionization and mass spectrometry	HPLC–PDA–ESI–MS/MS	[20]
High-performance liquid chromatography with electrospray ionization quadrupole time-of-flight and mass spectrometry	HPLC-ESI-Q-TOF-MS	[22]
High-performance liquid chromatography with diode array detectors	HPLC-DAD	[4,7]
Attenuated-total-reflectance mid-infrared spectroscopy	(ATR-IR)	[28]
Near-infrared spectroscopy	(NIR)	[28]

The authors declare that ATR-IR and NIR spectroscopy in combination with multivariate analysis is suitable for quantification of the baicalin and total baicalein content in SR, and it was found that ATR-IR spectroscopy provides higher accuracy in the given application. Moreover, a simple method for the simultaneous determination of four bioactive components, including baicalin, in composite preparations by microemulsion CE with UV detection at 273 nm was published recently [29]. The effect of the microemulsion addition ratio as well as borax and acetonitrile concentration, on the separation process, was evaluated. A running buffer composed of acetonitrile (8%) and 4% of microemulsion (consisting of 3.24% of n-heptane, 13.24% of sodium dodecyl sulfate (SDS), 26.44% of n-butanol, and 57.08% of distilled water) and 20 mM borax solution was found to be the most suitable for this separation. The LODs for four analytes were in the range of 0.50–1.2 μg/mL. In the tested concentration range, linear relationships between the peak areas and the concentrations of the analytes were obtained. The correlation coefficient for baicalin was 0.997.

#### 1.1.2. Electrochemical and Fluorescent Sensors

Most of the methods mentioned above provide good detection results for baicalin, but their application is limited by sophisticated pretreatment processes and time and cost-consuming operations. A good alternative may be the use of biosensors. Some reports state that electrochemical sensors designed for baicalin detection were characterized by a simple procedure, high sensitivity, and low time and cost constraints. Various functional materials, especially nanomaterials, have been used to modify the electrode in order to improve its sensitivity and selectivity. Ran et al. [30] described a highly sensitive electrochemical sensing platform based on the disulfide-linked β-cyclodextrin dimer (SS-β-CD) and ultrafine Pd clusters monodispersed on the surface of reduced graphene oxide (Pd@RGO). Due to the synergistic effects of the Pd@RGO and SS-b-CD, the SS-β-CD–Pd@RGO nanohybrid-modified electrode was found to have a linear response in the range of 0.02–20.00 mM for baicalin and of 0.01–10.00 mM for luteolin, with relatively low detection limits of 0.0052 mM and 0.0070 mM, respectively. This sensor was used to detect baicalin and luteolin in human serum samples using standard addition methods. Another electrochemical sensor with cyclodextrin moiety was described by Lio et al. [31]. In this case, the glassy carbon electrode (GCE) was modified with 2,6-dimethyl-β-cyclodextrin (DM-β-CD) functionalized graphene nanosheets (DM-β-CD-GNs), and the synergetic effects of GNs and DM-β-CD molecules induced increasing of the peak currents of baicalin and isoquercetin. The linear response ranges for isoquercetin and baicalin are 10 nM–3.0 μM and 0.04–3.0 μM with the LODs of 4 nM and 10 nM, respectively.

Sheng et al. [32] described a one-pot synthesis of Co nanoparticles (NPs) doped amino-graphene nanocomposites (Co-amino-Gr). By combining the merits of amino-Gr and the Co NPs, a highly sensitive electrochemical sensor was achieved. The proposed modified glass carbon electrode (Co-amino-Gr/GCE) exhibited a high specific voltametric response to baicalin. The response peak currents were linearly related with baicalin concentrations in the range of 1.0 × 10^−5^–8.0 × 10^−7^ mol/L with a LOD of 5.0 × 10^−9^ mol/L (S/N = 3). Additionally, the proposed method was used to detect baicalin in the medicinal capsules with satisfactory results. A voltammetry sensor platform for baicalein and baicalin simultaneous detection in vivo has also been developed and described [33]. The bimetallic oxide particles Ta_2_O_5_-Nb_2_O_5_@CTS composite modified directly in an antiseptic chitosan-modified carbon paste electrode was applied to the precise quantitative analysis of baicalein and baicalin for the first time. The linear detection range and limit of baicalein and baicalin on Ta_2_O_5_-Nb_2_O_5_@CTS-CPE were 0.08–8.0 μM for both of them and of 0.05 and 0.03 μM (S/N = 3), respectively. There are also reports on the usage of molybdenum sulfides as modifiers of GCE to obtain useful sensors and to develop a sensitive and selective electrochemical method for the determination of baicalin [34,35]. Baicalin exhibits enhanced voltametric response on the molybdenum disulfide (MoS_2_) nano-sheets-modified glassy carbon electrode (GCE) [34]. The electrochemical behavior of baicalin was investigated by cyclic voltammetry (CV) in phosphate-buffered saline (PBS) solution (pH 7.0) and by using differential pulse voltammetry (DPV). Under the optimized conditions, the oxidation peak current was linearly proportional to the baicalin concentrations in the range of 1.25 × 10^−7^–1.25 × 10^−5^ M, and the detection limit (S/N = 3) was calculated to be 5.0 × 10^−8^ M. In the other report, amorphous molybdenum sulfide (a-MoS_x_) nanocomposite based on biochar microsphere (BM) was prepared by the green and efficient hydrothermal method, and the discarded inedible pomelo peel was selected as the precursor of BM [35]. An a-MoS_x_-BM nanocomposite-modified GCE electrode was employed as a voltametric sensing platform for baicalin due to the synergistic effect of both BM and a-MoS_x_. The nanocomposite displayed an excellent linear sensing performance range from 10 nM to 5μ M for detecting baicalin with a low LOD of 2 nM. Moreover, an artificial intelligence technology based on an ML model was implemented to establish a smart sensing analysis platform by investigating the relationship between peak currents and analyte concentrations for smart analysis of baicalin in real samples.

An electrochemical sensor based on the FeO_x_/Fe@porous carbon composite (Fe@C) was also applied to the accurate and rapid determination of baicalin [36]. It occurred that Fe@C pyrolyzed at 800 °C had the optimum performance. The sensor Fe@C-800/ GCE has a linear range of 4–700 nM with a low LOD of 1.16 nM for the detection of baicalin in several natural plant samples and herbal medicine samples.

Fluorescent (FL) sensors for detecting baicalin were described recently [37,38]. A facile and efficient semi-quantitative method for versatile FL visual detection, which can promote the development of advanced chemo/biosensors and methods of analysis, was established [37]. A novel and versatile ratiometric FL biosensor based on the assembled nanohybrids of black phosphorus quantum dots (BPQDs)-doped metal-organic frameworks (MOF) and silver nanoclusters (AgNCs) and the enzyme-catalyzed reaction was constructed. The resultant biosensor has highly sensitive and selective ratiometric FL responses on baicalin. It allows the detection of baicalin in the range of 0.01–500 μg/mL, with a LOD of 3 ng/mL. This biosensor has high sensitivity, selectivity, and stability for baicalin detection in practical samples. A fluorescent sensor based on the inner filter effect (IFE) was successfully established using synthesized nitrogen-doped fluorescent carbon dots (N-CDs) to detect the content of baicalin in the Baicalin capsule and the quality of Scutellariae Radix [38]. The baicalin linear range of the method is 1.6–72 μg/mL (r = 0.9992), the LOD is 1.0 μg/mL, and the precision was 0.2%.

Furthermore, baicalin, along with other compounds from TCMs, were found to be new tyrosinase (TYR) inhibitors [39] or potential lipoxidase and superoxide dismutase inhibitors [40]. A combination of ligand fishing and the fluorescent enzymatic assay based on dopamine-functionalized carbon quantum dots (CQDs-Dopa) and a combination method comprising UF-LC-MS and HSCCC were used as identification methods for baicalin. Reports concerning profiling and determination of baicalin metabolites have also been published [41,42].

## 2. Pharmacological Effects of Baicalin

**Figure 2:**
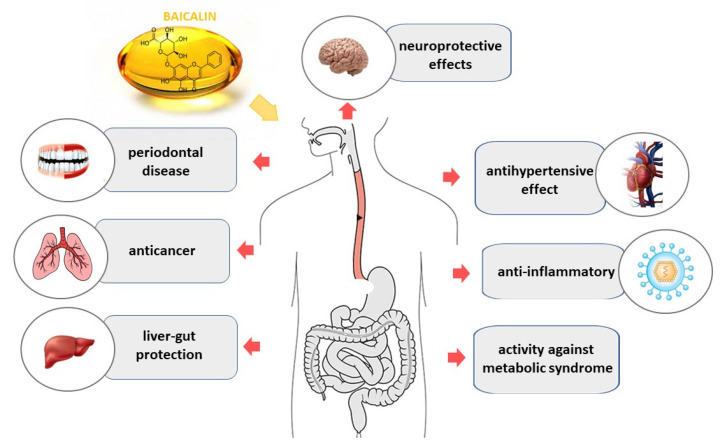
Selected pharmacological effects of baicalin (**1**) in humans.

Pharmacological studies have shown that SB and its components have a wide range of pharmacological activities, such as anti-inflammatory, antibacterial, antiviral, anticancer, antihypertensive, liver protection, stroke management, cardiovascular diseases treatment, etc. [43,44,45,46,47] (Figure 2).

Six flavones, including baicalin, proved to be the major bioactive flavones in SR. They possess great anti-inflammatory, anticancer, and antiviral effects [48,49]. The molecular mechanisms underlying the chemopreventive and chemotherapeutic applications of baicalin and baicalein in the treatment of cancer and inflammatory diseases were described recently [50]. Authors of the latest review presented in detail the anti-inflammatory effects of baicalin and baicalein on respiratory ailments, arthritis, type-2-diabetes and obesity, cardiovascular ailments, liver and neurodegenerative diseases and microbial infections, as well as the protective effect of them on cancer, including induction of apoptosis in cancer cells, suppression of metastasis and triggering of autophagy and cell cycle arrest in cancer cells. In general, baicalin exhibits these and many other pharmacological effects, which have been widely described and reviewed over the last decade, as will be presented in the following section.

The effect of baicalin on mitochondrial function and dynamics [51], potentially therapeutic effects in ocular disorders [52], neurogenerative diseases (due to its neuroprotective and cognitive enhancement potentials) [53], as well as in inflammatory disorders [54] have been thoroughly reviewed recently. More recent reviews regarding the antiviral properties [55] of baicalin and its regulatory action in cardiovascular diseases [56] have been reported. The role of intestinal microbiota in the pharmacokinetic characteristics of baicalin and baicalein, with regard to their pharmacological and toxicological effects, has also been presented [57].

The biological properties of baicalin, such as anti-inflammatory, antitumor, and antivirus ones, are related mainly to the regulatory effect of baicalin on the host immune response. Therefore an overview of its regulatory effect on toll-like receptors (TLRs) signaling pathways under various pathological conditions has also been provided [58]. Baicalin may have therapeutic effects on several well-known diseases and disorders, including cancer, ischemia, hypertension, etc. (Table 2).

### 2.1. Anticancer Effect

Last year, a study that summarized potential anticancer action of have been published, and the difference in their anticancer effects in animal models, as well as combination therapy and clinical trials, were compared [59]. The authors presented in detail the research results concerning breast, lung, colorectal, cervical, and liver cancers, and osteosarcoma and concluded that both baicalin and baicalein are effective in cancer. However, deglycosylated baicalin was found to have stronger anticancer potential. Therapeutic potentials of both the above-mentioned components to hematological cancer cell lines have also been explored [60]. Their activity in vitro towards leukemia, lymphoma, and myeloma cells was presented. For solid tumors that contain a huge mass of malignant tumors other than hematological malignancies, the in vivo pieces of evidence supporting the therapeutic potential of baicalin and baicalein have been reported, as well [61]. The role of baicalin as a regulator of cancer signaling pathways in cancer cell lines and as a mediator of anticancer efficacy in animal models, as well as the effectiveness of baicalin in combination therapy and in nano-formulations, have also been thoroughly reviewed [62]. Additionally, the usage of baicalin and other natural flavones in SB as potential medicinal drugs for the treatment of nicotine-induced non-small-cell lung cancer has been mentioned recently [63].

### 2.2. Ischemia

The neuroprotective effects of baicalin and baicalein in ischemia or stroke-induced neuronal cell death have been reported in 2017 [64]. All important information regarding the neuroprotective effect of baicalin and baicalein and their pharmacological mechanisms in various in vivo and in vitro experimental models of ischemic neuronal injury have been collected. The authors concluded that baicalin and baicalein are multi-target neuroprotective agents. This action is related to antioxidant, anti-apoptotic, anti-inflammatory, and anti-excitotoxic activity, protection of mitochondria, promotion of neuronal protective factors expression and adult neurogenesis, and also to other factors. Recently, Pan et al. [65] reviewed the promising pharmacological capabilities of baicalin, baicalein, and wogonin in preventing cell and tissue damage. Therapeutic potentials of the compounds against ischemia-induced neurotoxicity and damage in the brain and retina, in particular, in vitro findings on various brain cell types, in vivo findings on animal models, and their performance on brain ischemia models, have been emphasized. The authors suggested using the potential of these substances to develop new natural neuroprotective agents. Moreover, Bai et al. [66] demonstrated that baicalin significantly improved cardiac function decreased the myocardial infarction area, inhibited myocardial cell apoptosis, exerted a protective effect on cardiac microvessels, promoted the production of nitric oxide (NO) and elevated the level of cGMP in rats that underwent myocardial ischemia-reperfusion (IR) injury. It was concluded that baicalin protected cardiac microvascular endothelial cells (CMECs) in IR rats by promoting nitric oxide release via the PI3K-AKT-eNOS pathway and mitigated necroptosis by inhibiting the expression of RIP1, RIP3, and p-MLKL protein kinases. The authors claim that their study provides evidence that baicalin may serve as a potential therapeutic agent for CMEC protection in ischemic diseases. On the other hand, Hu et al. [67] showed that baicalin was effective in the treatment of myocardial ischemia (RI), myocardial infarction (MI), and IR injury on the basis of preclinical meta-analysis. Meta-analyses of cardiac pathology and function parameters, myocardial injury markers, and other indicators were performed. Potential mechanisms were categorized and summarized. Dose-response interval analyses were used to analyze the dose-response relationship between baicalin and myocardial ischemia disease. Fourteen studies and 222 animals were involved in the analysis. The authors declare that the therapeutic mechanism of baicalin action is related to a large number of pathways of anti-inflammatory, antioxidant, and anti-apoptotic activities, including regulation of ILs, JAK/STAT, TNF-α, NF-κB, PI3K/Akt, MAPK, and P2 × 3 pathways. The distribution dose of baicalin (in the analyses of the dose-effect relationship) is between 1 and 200 mg/kg. Baicalin exhibits positive effects on myocardial ischemia diseases, especially when the dose is within the range of 100–150 mg/kg.

### 2.3. Hypertension

**Figure 3:**
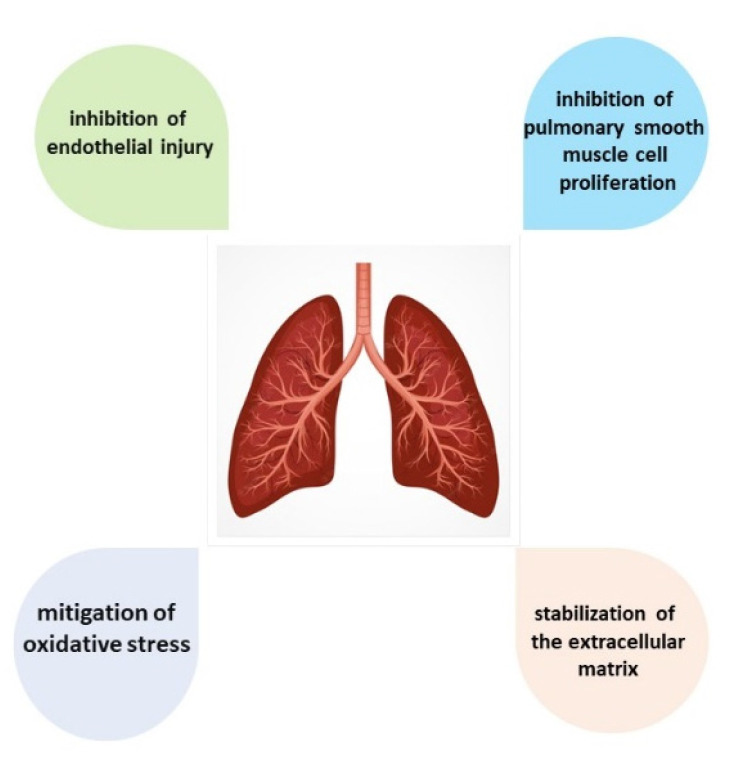
The baicalin action in the pulmonary system.

A comprehensive review regarding mechanistic and therapeutic aspects of baicalin and baicalein action in pulmonary hypertension (PH) has been published recently [68]. The authors have summarized the potential mechanisms that are responsible for the beneficial effects of baicalin and baicalein on PH, including anti-inflammatory response, inhibition of pulmonary smooth muscle cell proliferation, inhibition of endothelial injury and EndMT, stabilization of the extracellular matrix, mitigation of oxidative stress (Figure 3). Both in vivo and in vitro experiments that showed an effect on PH were demonstrated. However, the mechanism of their action needs further elucidation.

It was also reported that baicalin and berberine were the main antihypertensive constituents of Sanoshashinto herbs, and both exhibited the same antihypertensive effect [47]. Ding et al. [69] have recently demonstrated that the treatment with baicalin lowers the blood pressure in spontaneously hypertensive rats (SHRs) in vivo. Ex vivo vascular reactivity assay showed that baicalin relaxes phenylephrine (PE)-constricted SHR aortas, and this vasorelaxant effect of baicalin in SHR aortas is an endothelium-independent process. In addition, baicalin attenuated PE, Ang II, and KCl-induced vasoconstriction in SHR aortas. Intracellular Ca^2+^ regulation in vascular smooth muscles was mechanistically implicated in the vasorelaxant effect of baicalin under hypertensive conditions. Most notably, this effect of baicalin is partly dependent on activated K_ATP_ channels. Liu et al. [70] have also investigated the antihypertensive effects of baicalin and its molecular mechanisms. The authors demonstrated that baicalin treatment attenuates Ang II-induced elevation of blood pressure, vascular dysfunction, and pathological changes. Moreover, baicalin pretreatment attenuated Ang II-induced intracellular Ca^2+^ release, Angiotensin II type 1 receptor (AT1R) expression, and activation of MLCK/p- MLC signaling pathway in vascular smooth muscle cells (VSMCs).

### 2.4. Liver-Gut System

**Figure 4:**
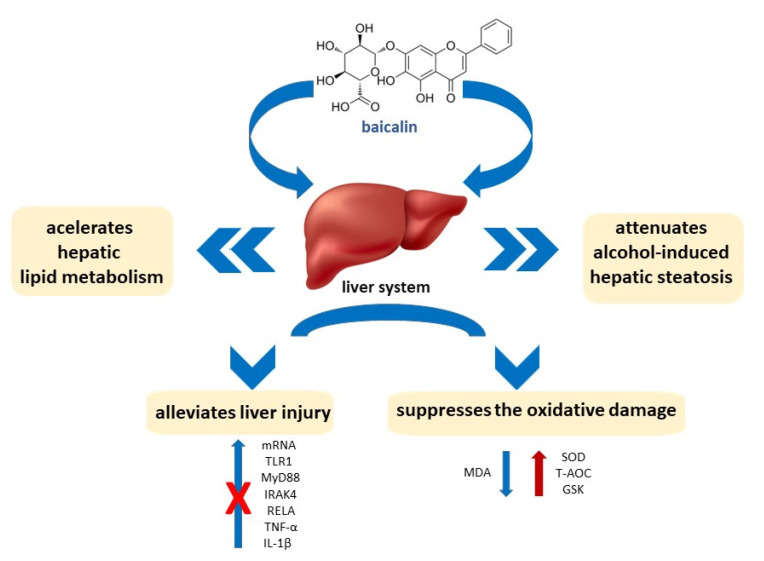
The effects of baicalin on the liver.

Two comprehensive reviews regarding the pharmacological effects of baicalin on liver diseases were published in 2021. Hu et al. [71] conducted the study on baicalin to determine the mechanism by which it regulates bile acid metabolism, intestinal flora, and related signaling pathways, providing new insights into the pharmacological effects of this compound. Liver and gut-related diseases’ protective activity of baicalin and its possible connection to liver-gut system diseases, as well as information on the protective effects of baicalin on the liver and gut, have been described in detail. The authors demonstrated that baicalin plays a therapeutic role mainly by mediating downstream apoptosis and immune response pathways induced by upstream oxidative stress and inflammation. At the same time, Yang et al. [72] summarized the pharmacological effects of baicalin to clarify its potential use in the treatment of liver diseases. They described the progress in the research on this subject and the mechanisms underlying the treatment of various liver diseases with baicalin in order to promote its clinical application. A wide range of pharmacological effects of baicalin, such as antioxidant, antiviral, anti-inflammatory, anti-obesity, and antitumor ones, as well as the main pharmacological mechanisms of baicalin action in liver diseases, have been demonstrated (Figure 4). The role of baicalin as a potential therapeutic agent in hepatobiliary and gastrointestinal disorders, including fatty liver syndrome, liver injury, liver fibrosis, cholestasis, hepatitis, HCC, colorectal cancer, and inflammatory bowel disease, as well as its interactions with intestinal microbiota has been extensively described by Ganguly et al. [73]. Additionally, the effects of dietary baicalin supplementation in Genetically Improved Farmed Tilapia (GIFT tilapia) have also been presented [74]. The pretreatments with baicalin effectively alleviated H_2_O_2_-induced liver injury. Pretreatments with 0.8 and/or 1.6 g/kg of baicalin suppressed the oxidative damage induced by H_2_O_2_, by increasing the levels of SOD, T-AOC, and GSH and decreasing the level of MDA, in both serum and liver. What is more important, pretreatments with 0.4, 0.8, and/or 1.6 g/kg of baicalin resulted in blocking the upregulation of the mRNA levels of TLR1, MyD88, IRAK4, RELA, TNF-α and IL-1β in THE H_2_O_2_-induced liver injury. The results indicated that dietary supplementation with baicalin increased feed efficiency, enhanced antioxidative ability, and alleviated liver damage in tilapia.

Li et al. [75] provided evidence to support the fact that baicalin attenuates alcohol-induced hepatic steatosis by activating hepatic lipolysis via suppressing SREBP1c elicited PNPLA3 competitive binding to ATGL and accelerating hepatic lipid metabolism. The results showed that baicalin at a dose of 200 mg/kg significantly attenuated the development of metabolic disorders and hepatic steatosis in alcohol-induced rats after four weeks of the treatment with alcohol (4 g/kg).

### 2.5. Metabolic Syndrome

Baicalin and baicalein have also been investigated for their activity against metabolic syndrome or disorders, and two reviews on this topic have appeared recently. Fang et al. [76] have focused on therapeutic applications and underlying molecular mechanisms of baicalin and baicalein against hyperglycemia, insulin resistance, type 2 diabetes, hyperlipidemia, obesity, and nonalcoholic fatty liver and tried to establish a novel anti-obese and antidiabetic strategy. Baicalin possesses hepatoprotective, anti-dyslipidemic, anti-lipogenic, anti-obese, and antidiabetic effects, being effective in treating obesity, insulin resistance, nonalcoholic fatty liver, and dyslipidemia. Effect of baicalin on obesity, insulin resistance, type 2 diabetes mellitus, and the antidiabetic and anti-dyslipidemic effects of baicalin along with the molecular mechanisms for its preventive and therapeutic applications in the treatment of insulin resistance, obesity, and diabetes have been shown. Rahimi et al. [77] evaluated different studies on the effect of SB and its two major bioactive constituents: baicalin and baicalein, on the critical factors of metabolic syndrome, including diabetes, hyperlipidemia, obesity, hypertension, and atherosclerosis. The authors evaluated mechanistically various possible signaling pathways involved in the pathogenesis of the metabolic syndrome. It occurred that baicalin and baicalein, two active constituents of *Scutellaria baicalensis*, by activation and upregulation of AMPK and PPAR-γ as the main signaling in the hemostasis of glucose and lipid metabolisms, may be promising candidates for the prevention and treatment of the metabolic syndrome. Antidiabetic properties of baicalin, baicalein, and SB, along with their metabolic effects and mechanisms of action, were summarized.

### 2.6. Protective Agent

**Figure 5:**
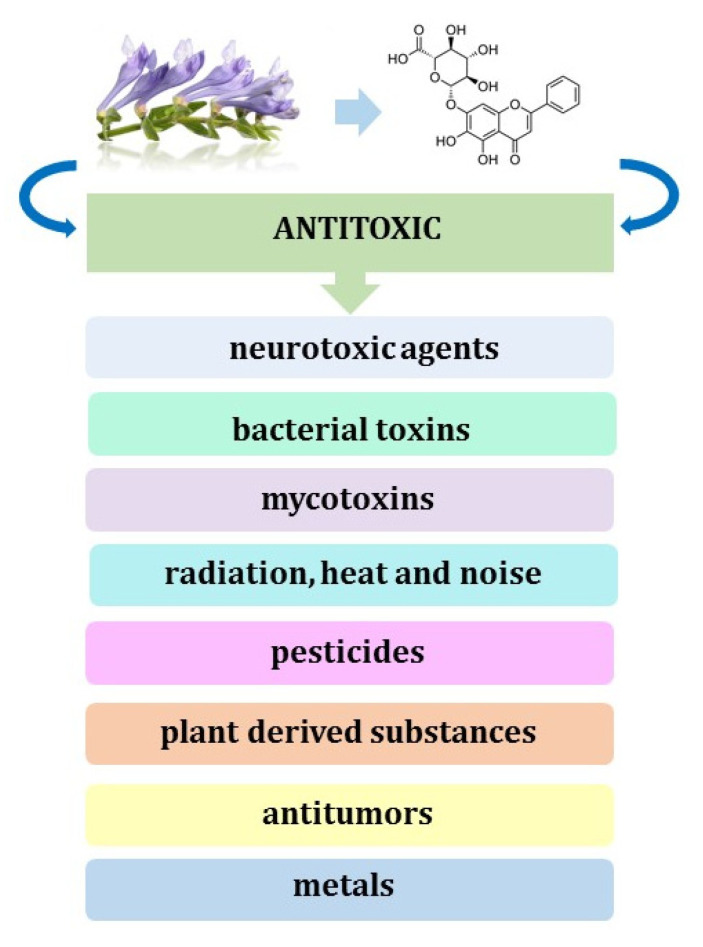
Antitoxic effects of baicalin (**1**).

Different studies demonstrated that baicalin, baicalein, and SB could act as potential antidotes or protective agents against the damage induced by natural toxins and physical factors through the alleviation of oxidative stress and inflammation [78]. The authors of the review presented the molecular mechanisms of their antitoxic effects and concluded that cytoprotective properties of baicalin and baicalein in vivo and in vitro are the most potent when they are introduced into the culture medium or animal prior to toxic compounds. Administration of baicalin is efficient in preventing or counteracting the morphological and functional impairments that are induced by mycotoxins (deoxynivalenol, DON), bacterial toxins (α-hemolysin, panton-valentine leukocidin, Shiga-like toxins, and Lipopolysaccharide), and plant-derived substances (ricin, aristolochic acid, concanavalin A, antimycin A, aconitine, and monocrotaline) in the liver, kidney, heart and other organs (Figure 5).

Moreover, it is effective in the protection of these organs against damage due to physical factors, such as radiation, heat, and noise. The same authors have also gathered and described the knowledge on protective and therapeutic effects of SB, baicalin, and baicalein against health damage due to chemical compounds that can cause intoxication after acute or chronic exposure and seriously affect various vital organs of the body, including brain, heart, liver, and kidneys [79]. Baicalin is significantly effective in healing various chemical insults, either directly or after conversion to baicalein. It increases cell viability and decreases organ damage and mortality when administered to culture media and animals with different neurotoxic agents, including metals (Fe and Al), pesticides (rotenone), antitumors (cisplatin), and other drugs, such as nitroglycerine, corticosterone, colistin, and sevoflurane. Moreover, flavonoids contained in SB possess significant protective potentials against antineoplastics (doxorubicin and bleomycin), common drugs (acetaminophen and estrogen), metals (Cd, Si, and Pb), and pesticides (paraquat) toxicity in the liver and lungs. The protective effects of SB and its flavonoids are attributed mainly to their activity in increasing antioxidant enzyme activity, inhibiting lipid peroxidation, reduction of the activity of inflammatory cytokines, and suppressing the apoptosis pathway.

Literature reports on the influence of baicalin-zinc and baicalin-copper complexes on DON-challenged piglets are also interesting [80,81]. Dietary supplementation with 5 g/kg of baicalin-copper complex alleviated inflammatory responses and regulated the secretion of appetite-regulating hormones and growth-axis hormones in DON-challenged piglets. On the other hand, a 0.5% baicalin-Zn complex basal diet modulated inflammatory and hormone secretion in piglets after DON exposure. In both cases, supplementation with the baicalin complex changed the composition of the intestinal microbiota in piglets.

### 2.7. Periodontal Disease

Baicalin has been shown by Ming et al. to possess multiple pharmacological activities in periodontal tissues [82]. Bacteriological, zoological, and cytological studies on the effects of baicalin in periodontal disease pathogenesis were presented, and five aspects of its pharmacological properties and the related mechanisms were investigated, which include antibacterial effect, protective effect on periodontal tissues, regulatory effect on pro-inflammatory mediators and matrix metalloproteinases, and regulatory effect on the innate immune response. However, the underlying mechanisms have not been fully defined. The proliferative effect and the involvement of baicalin in bone metabolism using human cementoblast-lineage cells have been verified and published lately [83]. A significant concentration-dependent increase in osteoprotegerin (OPG) expression and a decrease in receptor activator of nuclear factor-kB ligand (RANKL) gene expression were noticed in human cementoblast cell line (HCEM) cells in the 0.01, 0.1, and 1 μM baicalin treated groups. The authors concluded that baicalin increased OPG expression in human cementoblast lineage cells via the Wnt/β-catenin signaling pathway in HCEM cells. Therefore, baicalin could be applied as a complementary drug for periodontal regeneration in the future. The same research group had previously reported that baicalin enhanced the osteogenic differentiation of HCEM cells through the Wnt/β-catenin signaling pathway in vitro [84] and that baicalin ingestion during experimental tooth movement in rats increased OPG expression, decreased the expression of RANKL and suppressed root resorption [85].

### 2.8. Other

The regulatory role of baicalin in the diverse behaviors of distinct stem cell populations, including embryonic, cancer, neural, and other stem cells, has also been reviewed [86]. Its therapeutic effect is to soothe several disorders associated with menopause, providing novel insights into the treatment and prevention of climacteric syndrome. The authors concluded that baicalin could be used to treat climacteric sickness and control stem cell proliferation, differentiation, and self-renewal and therefore be a new alternative for the treatment of climacteric syndrome patients.

Zhou et al. [87] hypothesized that baicalin might protect dopaminergic neurons and increase brain dopamine levels, thus serving as an effective novel treatment for attention deficit hyperactivity disorder (ADHD). The hypothesis is based on the discovery that baicalin can pass through the blood-brain barrier (BBB) and is associated with the striatum and substantia nigra, which are enriched with dopaminergic neurons. It is worth emphasizing that the effect of baicalin on the brain’s dopamine system has not been reported so far.

The therapeutic effects of baicalin on the recovery of vitiligo stimulated by monophenylketone in mice have also been reported [88]. The intraperitoneal injection with 2 mg of baicalin in 100 μL of 5% NaHCO_3_ aqueous solution has been performed every day. It was observed that baicalin slowed down the progression of depigmentation, decreased the incidence of depigmentation, and reduced the depigmentation area. Moreover, baicalin increased the number of epidermal melanocytes in depigmented skin, decreased CD8^+^ T cell infiltration in mice skin, decreased the expression of CXCL10 and CXCR3 and decreased the levels of serum cytokine (interleukin (IL)-6, tumor necrosis factor (TNF-α), interferon-*γ (*IFN-*γ)*, and IL-13).

The possible mechanism of the anti-UVB effect of baicalin in human skin fibroblasts (HSFs) has been recently proposed by Zhang et al. [89]. Baicalin exerted cytoprotective effects in UVB-induced HSFs. What is more, it increased autophagy and suppressed the UVB-induced apoptosis of HSFs. It occurred that baicalin has the ability to protect UVB-irradiated HSFs from apoptosis by inducing autophagy through the upregulation of AMPK phosphorylation and the downregulation of mTOR phosphorylation. In this study, baicalin was used at a concentration of 25 ng/mL.

The anti-migraine activity of baicalin and other active compounds of Duijinsan, the popular Chinese herbal medicine composed of *Radix scutellariae* and *Rhei Radix*, have also been examined [90]. The compounds were screened for their anti-migraine effect by the combination of two methods: spectrum-effect relationship analysis and molecular docking. In vitro validation experiments showed that five compounds predicted to be active, including baicalin, inhibited the calcitonin gene-related peptide (CGRP) release and inhibited activation of the TRPV1 channel. The effect of these compounds on trigeminal nerve cells releasing CGRP stimulated by tumor necrosis factor-α (TNF-α) showed that they inhibited the release of CGRP caused by TNF-α stimulation in a dose-dependent manner. At 270 μmol/L, compared with the model group, all the predicted compounds significantly decreased the CGRP level, proving that the compounds had certain in vitro anti-migraine activity. Moreover, baicalin, chrysin-7-O-β-D-glucuronide, and oroxylin A-7-glucoronide significantly inhibited the activation of the TRPV1 channel and were speculated to be the key active components.

The use of baicalin as a potential therapeutic option for the treatment of renal interstitial fibrosis (RIF) by possibly inhibiting the TGF-β/Smad signaling pathway has been reported recently [91]. The unilateral ureteral obstruction (UUO) model of RIF was constructed and treated with 10, 20, and 40 mg/kg of baicalin doses or with 8 mg/kg of valsartan as a positive control. Increased levels of p-Smad2 and p-Smad3 were observed in UUO mice and in TGF-β1-treated myofibroblasts. In contrast, baicalin treatment significantly decreased p-Smad2, p-Smad3, and TGF-β protein expression. The collected data suggested that baicalin treatment inhibited the activation of the TGF-b/Smad pathway both in UUO mice and in TGF-β1-treated myofibroblasts.

### 2.9. In Vivo Studies

There are also many proofs of the health-promoting properties of baicalin, determined in vivo.

Novel immunopharmacological functions of baicalin in treating recurrent spontaneous abortion (RSA) have also been investigated lately [92]. In an in vivo experiment, baicalin at the doses of 25, 50, and 100 mg/kg in 200 μL of normal saline was administered to RSA mice from day 0.5 of pregnancy until day 12.5. It has been proved that baicalin protected mice from RSA by reversing conventional dendritic cells (DCs) to plasmacytoid DCs, and the expression of DC-related functional molecular MHC-II/HLA-DR, CD80, CD86, CD274, and 33D1 through the regulation of the STAT5-ID2/E2-2 pathway.

Network pharmacology is a new discipline that integrates systems biology, network biology, computational biology, multidirectional pharmacology, molecular pharmacology, molecular dynamics, and other multidisciplinary technologies. In the study by Chen and co-workers, network pharmacology was used to help in exploring the potential mechanism of action of baicalin in the treatment of hair loss [93]. One hundred eighty-nine potential targets of baicalin for the treatment of alopecia were predicted in this study, with 122 overlapping targets with alopecia, indicating that baicalin has multiple targets in this treatment. It was observed that baicalin at the concentration of 0.1 μg/mL exerted a significant effect on HHDPC proliferation. Baicalin was capable of inducing the phosphorylation of AKT and activating IGF1 and ALP and, by that, promoting hair growth.

**Table 2:** The pharmacological aspects of baicalin.

Pharmacological Effects	Refs.
Anticancer effect	[48,59,60,61,62,63,73]
Ischemia	[64,65,66,67]
Hypertension	[47,68,69,70]
Liver-gut system	[71,72,73,74,75]
Metabolic syndrome	[76,77]
Protective agent	[78,79,80,81]
Periodontal disease	[82,84,85]
Anti-inflammatory effects	[48,50,54,72]
Antiviral properties	[48,55,72]
Other	[86,87,88,89,90,91,92,93]

After oral administration of baicalin to BALB/c mice infected with the influenza A/FM1/1/47(H1N1) virus, its anti-influenza effect was observed [94]. Similar conclusions were drawn by Nayak et al. [95], who confirmed the antiviral activity of baicalin against influenza strain A/H1N1/Eastern India/66/pdm09 (H1N1-pdm09). At the same time, Liu et al. [96] studied the mechanism underlying the anti-depressant activity of baicalin. Administration of this drug to mice at doses of 25 and 50 mg/kg considerably decreased levels of inflammatory cytokines IL-1β, IL-6, and TNF-α and inhibited the expressions of HMGB1, TLR4, and p-NF-κBp65. Moreover, hepatoprotective effects of baicalin (at a dose of 10 mg/kg) were observed in mice with CCl_4_-induced liver injuries. However, no significant effect on APAP-induced intoxication was noticed [97]. Additionally, there is research on new, more effective delivery methods aiming to increase the bioavailability of baicalin after oral administration, using mixed micelles containing Pluronic P123 copolymer (P123) and sodium taurocholate (ST) as carrier materials (in vivo study—rat model) [98].

According to the most recent research, baicalin can inhibit the proliferation of airway smooth muscle cells (ASMC) through the rat sarcoma viral oncogene (RAS) signaling pathway. The study was performed on a mouse model with bronchial asthma induced by ovalbumin (OVA) [99]. At the same time, another research team confirmed that baicalin ameliorated the symptoms of nonalcoholic fatty liver disease (NAFLD) induced in mice by a high-fat diet (HFD) through AMPK-mediated inhibition of SREBP1 and NF-κB pathways and activation of Nrf2 pathway [100]. Topical application of baicalin (100 mg/mL) in a mouse model of pressure ulcers accelerates wound healing through the downregulation of pro-inflammatory cytokines (IL-6 and IL-1β) along with the upregulation of the anti-inflammatory cytokine IL-10 and several growth factors (VEGF, FGF-2, PDGF-β, and CTGF) [101]. Furthermore, baicalin ameliorates corticosterone-induced depression symptoms by promoting the neurodevelopment of the hippocampal via mTOR/GSK3 β pathway [102]. There are also some pieces of evidence that baicalin exerts anti-depressant effects by increasing the expression of genes encoding enzymes involved in the glycolytic pathway and TCA cycle, thus improving brain energy and function [103]. In addition, it has been found that baicalin inhibits oxidative injuries and apoptosis in uterine tissue, induced by acute heat stress, which in the future may find application in the treatment of infertility by the methods aiming to accelerate embryo implantation [104]. What is more, baicalin has the potential to treat acute graft-versus-host disease (aGVHD), which is the main complication of and cause of death after allogeneic hematopoietic stem cell transplantation [105].

To sum up, over the years, there has been growing interest in health-promoting substances isolated from natural sources, including the roots of medicinal plants. Numerous in vitro studies, which were conducted for many years, recently have been confirmed on animal models, mostly on mice and swine. There has been no information on the harmful effects of baicalin so far. Nevertheless, it is necessary to carry out thorough clinical research in order to unequivocally eliminate any possible side effects of this drug.

## 3. Conclusions

Baicalin, being a natural compound isolated from *Scutellaria baicalensis*, has a high therapeutic value. Therefore the development of new sensitive and selective detection methods of high accuracy is an important task. Generally, methods for the detection of baicalin are based on liquid chromatography (HPLC) separations in combination with mass spectrophotometry, diode array detector, or photodiode-array detector. However, there are also other separation techniques used for this purpose, such as thin layer chromatography, capillary electrophoresis, and micellar electrokinetic capillary chromatography. Most recent methods are based on biosensors with FeOx/Fe@porous carbon composite and molybdenum sulfide nanocomposite, prepared by the green method. Additionally, fluorescent sensors based on the assembled nanohybrids of black phosphorus quantum dots and silver nanoclusters are considered a facile and efficient quantitative method. It is worth mentioning that baicalin has a very wide spectrum of biological activities, including antihypertensive, neuroprotective, and antidiabetic, and might be used to treat hypertension and ischemia and to prevent liver diseases. The data presented above could be an inspiration for further study on discovering better detection methods for baicalin, providing higher accuracy and selectivity, and for future investigation of potential pharmaceutical use of baicalin and elucidation of molecular mechanisms of its action.

## Data Availability

Data sharing not applicable.

## Abbreviations

AgNCs	silver nanoclusters
AMPK	5′AMP-activated protein kinase
Ang II	angiotensin II
ATGL	adipose triglyceride lipase
ATR-IR	attenuated-total-reflectance mid-infrared
CMECs	cardiac microvascular endothelial cells
DAD	diode array detectors
DM-β-CD	2,6-dimethyl-β-cyclodextrin
DON	deoxynivalenol
DPV	differential pulse voltammetry
GCE	glassy carbon electrode
HPLC	high-performance liquid chromatography
HSCCC	high-performance countercurrent chromatography
LC-MS/MS	liquid chromatography–tandem mass spectrometry
IR	ischemia-reperfusion
LC-UV	liquid chromatography with ultraviolet detector
LOQ	limit of quantification
LOT	limit of detection
MAPK	mitogen-activated protein kinase
MEKC	micellar electrokinetic capillary chromatography
MI	myocardial infarction
MS	mass spectrometry
NAFLD	nonalcoholic fatty liver disease
NIR	near-infrared spectroscopy
NF-κB	nuclear factor kappa-B
NO	nitric oxide
OPG	osteoprotegerin
UHPLC	ultra-high-performance liquid chromatography
UUO	unilateral ureteral obstruction
PE	phenylserine
PH	pulmonary hypertension
PDA	photodiode-array detectors
ROS	reactive oxygen species
RSD	relative standard deviation
SDS	sodium dodecyl sulfate
SHRs	spontaneously hypertensive rats
SOD	sphincter of oddi dysfunction
SREBP1c	sterol regulatory element-binding protein 1c
TLC	thin layer chromatography
TLRs	toll-like receptors
TNF-α	tumor necrosis factor α
TYR	tyrosinase
VSMCs	vascular smooth muscle cells

## References

[1] Zhao Q., Chen X.Y., Martin C. (2016). *Scutellaria baicalensis*, the Golden Herb from the Garden of Chinese Medicinal Plants. Sci. Bull..

[2] Wang H.-Z., Yu C.-H., Gao J., Zhao G.-R. (2007). Effects of Processing and Extracting Methods on Active Components in Radix Scutellariae by HPLC Analysis. Zhongguo Zhongyao Zazhi.

[3] Seo C.S., Shin H.K. (2021). Development of a Simultaneous Analysis Method for Quality Control of a Traditional Herbal Formula, Daeshiho-Tang, Using 10 Marker Components. Appl. Sci..

[4] Chen J.C., Wu H.L., Wang T., Dong M.Y., Chen Y., Yu R.Q. (2022). High-Performance Liquid Chromatography–Diode Array Detection Combined with Chemometrics for Simultaneous Quantitative Analysis of Five Active Constituents in a Chinese Medicine Formula Wen-Qing-Yin. Chemosensors.

[5] Zhang Y., Ding Y., Zhang T., Jiang X., Yi Y., Zhang L., Chen Y., Li T., Kang P., Tian J. (2019). Quantitative Analysis of Twelve Active Components Combined with Chromatographic Fingerprint for Comprehensive Evaluation of Qinma Prescription by Ultra-Performance Liquid Chromatography Coupled with Diode Array Detection. J. Chromatogr. Sci..

[6] Wu T.Y., Chang F.R., Liou J.R., Lo I.W., Chung T.C., Lee L.Y., Chi C.C., Du Y.C., Wong M.H., Juo S.H.H. (2016). Rapid HPLC Quantification Approach for Detection of Active Constituents in Modern Combinatorial Formula, San-Huang-Xie-Xin-Tang (SHXXT). Front. Pharmacol..

[7] Li B.Q., Chen J., Li J.J., Wang X., Zhai H.L., Zhang X.Y. (2015). High-Performance Liquid Chromatography with Photodiode Array Detection and Chemometrics Method for the Analysis of Multiple Components in the Traditional Chinese Medicine Shuanghuanglian Oral Liquid. J. Sep. Sci..

[8] Seo C.S., Shin H.K. (2016). HPLC-PDA Method for Simultaneous Determination of Nine Marker Components in Banhasasim-Tang. J. Chromatogr. Sci..

[9] Zhang Y.F., Lu Y., Xiao J., Chen Y., Wang X.H. (2020). Concurrent Identification of 11 Major Primary Active Compounds in Huangqin Qingfei Decoction by Liquid Chromatography Tandem Mass Spectrometry via Liquid Chromatography Tandem Mass Spectrometry. Pak. J. Pharm. Sci..

[10] Wang Z., An R., Du G., Liang K., Li G. (2019). Validation of an LC–MS/MS Method for Simultaneous Detection of Diverse Components of Qinxing Qingre Zhike Granule in Rat Plasma and Its Application to Pharmacokinetic Study after Oral Administration to Rats. Biomed. Chromatogr..

[11] Wang Y., Zhang Y., Xiao J., Xu R., Wang Q., Wang X. (2018). Simultaneous Determination of Baicalin, Baicalein, Wogonoside, Wogonin, Scutellarin, Berberine, Coptisine, Ginsenoside Rb1 and Ginsenoside Re of Banxia Xiexin Decoction in Rat Plasma by LC–MS/MS and Its Application to a Pharmacokinetic Study. Biomed. Chromatogr..

[12] Zhang Y., Yuan J., Zhang Y., Chen Y., Cao J., An R., Wang X. (2015). LC-MS/MS Analysis of Gegen Qinlian Decoction and Its Pharmacokinetics after Oral Administration to Rats. Biomed. Chromatogr..

[13] Hu L., Xiong Y., Zou Z., Yang Y., He J., Zhong L., Wang Y., Yang M. (2020). Identifying the Chemical Markers in Raw and Wine-Processed *Scutellaria baicalensis* by Ultra-Performance Liquid Chromatography/Quadrupole Time-of-Flight Mass Spectrometry Coupled with Multiple Statistical Strategies. Biomed. Chromatogr..

[14] Zhang F., Li Z., Li M., Yuan Y., Cui S., Chen J., Li R. (2020). An Integrated Strategy for Profiling the Chemical Components of Scutellariae Radix and Their Exogenous Substances in Rats by Ultra-High-Performance Liquid Chromatography/Quadrupole Time-of-Flight Mass Spectrometry. Rapid Commun. Mass Spectrom..

[15] Baygildieva D.I., Baygildiev T.M., Stavrianidi A.N., Shpigun O.A., Rodin I.A. (2018). Simultaneous Determination of Wogonin, Scutellarin, Baicalin, and Baicalein in Extracts from *Scutellariae baicalensis* by High-Performance Liquid Chromatography with Tandem Mass Spectrometry. J. Anal. Chem..

[16] Cui X., Cai H., Li H., Tao Y., Huang P., Qian X., Li J., Cai B. (2016). Simultaneous Determination of 10 Flavonoids in Crude and Wine-Processed Radix Scutellariae by UHPLC. J. Chromatogr. Sci..

[17] Cui X.B., Qian X.C., Huang P., Zhang Y.X., Li J.S., Yang G.M., Cai B.C. (2015). Simultaneous Determination of Ten Flavonoids of Crude and Wine-Processed Radix Scutellariae Aqueous Extracts in Rat Plasma by UPLC-ESI-MS/MS and Its Application to a Comparative Pharmacokinetic Study. Biomed. Chromatogr..

[18] Li H., Jiang Y., Chen F. (2004). Separation Methods Used for *Scutellaria Baicalensis* Active Components. J. Chromatogr. B Analyt. Technol. Biomed. Life Sci..

[19] Luo J.L., Lu F.L., Liu Y.C., Lo C.F. (2012). Identification of *Scutellaria Baicalensis* in Traditional Chinese Medicine Preparations by LC/MS/MS Fingerprinting Method. J. Food Drug Anal..

[20] Shi G.F., Yao R.X., Wang G.Y., Wang Z.J., Chen F.W. (2015). Liquid Chromatography-Tandem Mass Spectrometry Screening Method for the Detection of Radical-Scavenging Natural Antioxidants from the Whole Scutellariae (Radix, Stem and Leaf). J. Chromatogr. Sci..

[21] Islam M.N., Chung H.J., Kim D.H., Yoo H.H. (2012). A Simple Isocratic HPLC Method for the Simultaneous Determination of Bioactive Components of Scutellariae Radix Extract. Nat. Prod. Res..

[22] Yan Z., Liqiong S., Yingduo Y., Jin Q., Boyang Y. (2020). Application of Multi-Dimensional and Multi-Informational (MD-MI) Integrated Xanthine Oxidase and Superoxide Anion Fingerprint in Quality Evaluation of Scutellariae Radix. J. Pharm. Biomed. Anal..

[23] Weiping L., Yukui R. (2010). Short Communication Evaluation of Baicalin in *Scutellaria baicalensis* georgi Using HPLC Method. Bull. Chem. Soc. Ethiop..

[24] Chen H., Li Z., Li Y., Wu X., Wang S., Chen K., Zheng X., Du Q., Tang D. (2015). Simultaneous Determination of Baicalin, Oroxylin A-7-O-Glucuronide and Wogonoside in Rat Plasma by UPLC-DAD and Its Application in Pharmacokinetics of Pure Baicalin, Radix Scutellariae and Yinhuang Granule. Biomed. Chromatogr..

[25] Wei Y., Pi C., Yang G., Xiong X., Lan Y., Yang H., Zhou Y., Ye Y., Zou Y., Zheng W. (2016). LC-UV Determination of Baicalin in Rabbit Plasma and Tissues for Application in Pharmacokinetics and Tissue Distribution Studies of Baicalin after Intravenous Administration of Liposomal and Injectable Formulations. Molecules.

[26] Pang H., Shi A., Li M., Xue W., Li Y., Cao G., Yan B., Dong F., Xiao W., He G. (2016). Simultaneous Determination of Baicalein and Baicalin in Human Plasma by High Performance Liquid Chromatograph-Tandem Spectrometry and Its Application in a Food-Effect Pharmacokinetic Study. Drug Res..

[27] Tu Y., Zhou L., Li L., Wang L., Gao S., Hu M. (2020). Development and Validation of an LC-MS/MS Method for the Quantification of Flavonoid Glucuronides (Wogonoside, Baicalin, and Apigenin-Glucuronide) in the Bile and Blood Samples: Application to a Portal Vein Infusion Study. Anal. Biochem..

[28] Navarro Escamilla M., Rodenas Sanz F., Li H., Schönbichler S.A., Yang B., Bonn G.K., Huck C.W. (2013). Rapid Determination of Baicalin and Total Baicalein Content in Scutellariae Radix by ATR-IR and NIR Spectroscopy. Talanta.

[29] Li C., Xu Y. (2022). Determination of Puerarin, Daidzein, Baicalin and Wogonin in Composite Preparations by Capillary Electrophoresis. J. Chem. Soc. Pak..

[30] Ran X., Yang L., Zhao G., Ye H., Zhang Y., Fan S., Xie X., Zhao H., Li C.P. (2015). Simultaneous Determination of Two Flavonoids Based on Disulfide Linked β-Cyclodextrin Dimer and Pd Cluster Functionalized Graphene-Modified Electrode. RSC Adv..

[31] Liu Z., Zhang A., Guo Y., Dong C. (2014). Electrochemical Sensor for Ultrasensitive Determination of Isoquercitrin and Baicalin Based on DM-β-Cyclodextrin Functionalized Graphene Nanosheets. Biosens. Bioelectron..

[32] Sheng K., Wang L., Li H., Zou L., Ye B. (2017). Green Synthesized Co Nanoparticles Doped Amino-Graphene Modified Electrode and Its Application towards Determination of Baicalin. Talanta.

[33] Xie Z., Lu W., Yang L., Li G., Ye B. (2017). A Voltammetry Sensor Platform for Baicalein and Baicalin Simultaneous Detection in Vivo Based on Ta_2_O_5_-Nb_2_O_5_@CTS Composite. Talanta.

[34] Zhang H., Wang T., Qiu Y., Fu F.F., Yu Y. (2016). Electrochemical Behavior and Determination of Baicalin on a Glassy Carbon Electrode Modified with Molybdenum Disulfide Nano-Sheets. J. Electroanal. Chem..

[35] Rao L., Zhou P., Liu P., Lu X., Duan X., Wen Y., Zhu Y., Xu J. (2021). Green Preparation of Amorphous Molybdenum Sulfide Nanocomposite with Biochar Microsphere and Its Voltametric Sensing Platform for Smart Analysis of Baicalin. J. Electroanal. Chem..

[36] Li J., Wang Y., Wang C., Wang Y., Yang Y., Chen J., Li C., Xie Y., Zhao P., Fei J. (2022). Uncovering the Optimal Pyrolysis Temperature of NH2-MIL-88B-Derived FeOX/Fe@porous Carbon Composites for the Ultrasensitive Electrochemical Detection of Baicalin in Natural Plant Samples. Carbon.

[37] Jiang X., Jin H., Sun Y., Sun Z., Gui R. (2020). Assembly of Black Phosphorus Quantum Dots-Doped MOF and Silver Nanoclusters as a Versatile Enzyme-Catalyzed Biosensor for Solution, Flexible Substrate and Latent Fingerprint Visual Detection of Baicalin. Biosens. Bioelectron..

[38] Cheng W., Wu S., Wang D., Lou Y. (2022). Detection of Baicalin Capsule and Scutellariae Radix Based on Nitrogen-Doped Carbon Dots as a Fluorescence Probe. Results Chem..

[39] Zhao Y., Hu J.J., Bai X.L., Liu H.P., Qi X.W., Liao X. (2022). Fast Screening of Tyrosinase Inhibitors from Traditional Chinese Medicinal Plants by Ligand Fishing in Combination with in Situ Fluorescent Assay. Anal. Bioanal. Chem..

[40] Xia J., Liu C., Niu H., Hou W., Li S. (2021). Screening and Isolation of Potential Lipoxidase and Superoxide Dismutase Inhibitors from *Scutellaria baicalensis* Georgi Using High-Speed Countercurrent Chromatography Target-Guided by Ultrafiltration-Liquid Chromatography-Mass Spectrometry. J. Sep. Sci..

[41] Lu Q.Y., Zhang L., Moro A., Chen M.C., Harris D.M., Eibl G., Go V.L.W. (2012). Detection of Baicalin Metabolites Baicalein and Oroxylin-a in Mouse Pancreas and Pancreatic Xenografts. Pancreas.

[42] Zhang J., Cai W., Zhou Y., Liu Y., Wu X., Li Y., Lu J., Qiao Y. (2015). Profiling and Identification of the Metabolites of Baicalin and Study on Their Tissue Distribution in Rats by Ultra-High-Performance Liquid Chromatography with Linear Ion Trap-Orbitrap Mass Spectrometer. J. Chromatogr. B Analyt. Technol. Biomed. Life Sci..

[43] Zhao T., Tang H., Xie L., Zheng Y., Ma Z., Sun Q., Li X. (2019). *Scutellaria baicalensis* Georgi. (Lamiaceae): A Review of Its Traditional Uses, Botany, Phytochemistry, Pharmacology and Toxicology. J. Pharm. Pharmacol..

[44] Tan Y.Q., Lin F., Ding Y.K., Dai S., Liang Y.X., Zhang Y.S., Li J., Chen H.W. (2022). Pharmacological Properties of Total Flavonoids in *Scutellaria baicalensis* for the Treatment of Cardiovascular Diseases. Phytomedicine.

[45] Song J.W., Long J.Y., Xie L., Zhang L.L., Xie Q.X., Chen H.J., Deng M., Li X.F. (2020). Applications, Phytochemistry, Pharmacological Effects, Pharmacokinetics, Toxicity of *Scutellaria baicalensis* Georgi. And Its Probably Potential Therapeutic Effects on COVID-19: A Review. Chin. Med..

[46] Gaire B.P., Moon S.K., Kim H. (2014). *Scutellaria baicalensis* in Stroke Management: Nature’s Blessing in Traditional Eastern Medicine. Chin. J. Integr. Med..

[47] Wu J., Nakashima S., Shigyo M., Yamasaki M., Ikuno S., Morikawa A., Takegami S., Nakamura S., Konishi A., Kitade T. (2020). Antihypertensive Constituents in Sanoshashinto. J. Nat. Med..

[48] Li C., Lin G., Zuo Z. (2011). Pharmacological Effects and Pharmacokinetics Properties of Radix Scutellariae and Its Bioactive Flavones. Biopharm. Drug Dispos..

[49] Bao M., Ma Y., Liang M., Sun X., Ju X., Yong Y., Liu X. (2022). Research Progress on Pharmacological Effects and New Dosage Forms of Baicalin. Vet. Med. Sci..

[50] Hu Z., Guan Y., Hu W., Xu Z., Ishfaq M. (2022). An Overview of Pharmacological Activities of Baicalin and Its Aglycone Baicalein: New Insights into Molecular Mechanisms and Signaling Pathways. Iran J. Basic Med. Sci..

[51] de Oliveira M.R., Nabavi S.F., Habtemariam S., Erdogan Orhan I., Daglia M., Nabavi S.M. (2015). The Effects of Baicalein and Baicalin on Mitochondrial Function and Dynamics: A Review. Pharmacol. Res..

[52] Xiao J.R., Do C.W., To C.H. (2014). Potential Therapeutic Effects of Baicalein, Baicalin, and Wogonin in Ocular Disorders. J. Ocul. Pharmacol. Ther..

[53] Sowndhararajan K., Deepa P., Kim M., Park S.J., Kim S. (2018). Neuroprotective and Cognitive Enhancement Potentials of Baicalin: A Review. Brain Sci..

[54] Dinda B., Dinda S., DasSharma S., Banik R., Chakraborty A., Dinda M. (2017). Therapeutic Potentials of Baicalin and Its Aglycone, Baicalein against Inflammatory Disorders. Eur. J. Med. Chem..

[55] Li K., Liang Y., Cheng A., Wang Q., Li Y., Wei H., Zhou C., Wan X. (2021). Antiviral Properties of Baicalin: A Concise Review. Rev. Bras. Farmacogn..

[56] Xin L., Gao J., Lin H., Qu Y., Shang C., Wang Y., Lu Y., Cui X. (2020). Regulatory Mechanisms of Baicalin in Cardiovascular Diseases: A Review. Front. Pharmacol..

[57] Noh K., Kang Y., Nepal M.R., Jeong K.S., Oh D.G., Kang M.J., Lee S., Kang W., Jeong H.G., Jeong T.C. (2016). Role of Intestinal Microbiota in Baicalin-Induced Drug Interaction and Its Pharmacokinetics. Molecules.

[58] Jiang M., Li Z., Zhu G. (2020). Immunological Regulatory Effect of Flavonoid Baicalin on Innate Immune Toll-like Receptors. Pharmacol. Res..

[59] Wang L., Feng T., Su Z., Pi C., Wei Y., Zhao L. (2022). Latest Research Progress on Anticancer Effect of Baicalin and Its Aglycone Baicalein. Arch. Pharm. Res..

[60] Chen H., Gao Y., Wu J., Chen Y., Chen B., Hu J., Zhou J. (2014). Exploring Therapeutic Potentials of Baicalin and Its Aglycone Baicalein for Hematological Malignancies. Cancer Lett..

[61] Gong W., Zhao Z.X., Liu B.J., Lu L.W., Dong J.C. (2017). Exploring the Chemopreventive Properties and Perspectives of Baicalin and Its Aglycone Baicalein in Solid Tumors. Eur. J. Med. Chem..

[62] Singh S., Meena A., Luqman S. (2021). Baicalin Mediated Regulation of Key Signaling Pathways in Cancer. Pharmacol. Res..

[63] Alsharairi N.A. (2021). *Scutellaria baicalensis* and Their Natural Flavone Compounds as Potential Medicinal Drugs for the Treatment of Nicotine-Induced Non-Small-Cell Lung Cancer and Asthma. Int. J. Environ. Res. Public Health.

[64] Liang W., Huang X., Chen W. (2017). The Effects of Baicalin and Baicalein on Cerebral Ischemia: A Review. Aging Dis..

[65] Pan L., Cho K.S., Yi I., To C.H., Chen D.F., Do C.W. (2021). Baicalein, Baicalin, and Wogonin: Protective Effects against Ischemia-Induced Neurodegeneration in the Brain and Retina. Oxid. Med. Cell Longev..

[66] Bai J., Wang Q., Qi J., Yu H., Wang C., Wang X., Ren Y., Yang F. (2019). Promoting Effect of Baicalin on Nitric Oxide Production in CMECs via Activating the PI3K-AKT-ENOS Pathway Attenuates Myocardial Ischemia–Reperfusion Injury. Phytomedicine.

[67] Hu S., Jiang L., Yan Q., Zhou C., Guo X., Chen T., Ma S., Luo Y., Hu C., Yang F. (2022). Evidence Construction of Baicalin for Treating Myocardial Ischemia Diseases: A Preclinical Meta-Analysis. Phytomedicine.

[68] Cui L., Yuan T., Zeng Z., Liu D., Liu C., Guo J., Chen Y. (2022). Mechanistic and Therapeutic Perspectives of Baicalin and Baicalein on Pulmonary Hypertension: A Comprehensive Review. Biomed. Pharmacother..

[69] Ding L., Jia C., Zhang Y., Wang W., Zhu W., Chen Y., Zhang T. (2019). Baicalin Relaxes Vascular Smooth Muscle and Lowers Blood Pressure in Spontaneously Hypertensive Rats. Biomed. Pharmacother..

[70] Liu H., Cheng Y., Chu J., Wu M., Yan M., Wang D., Xie Q., Ali F., Fang Y., Wei L. (2021). Baicalin Attenuates Angiotensin II-Induced Blood Pressure Elevation and Modulates MLCK/p-MLC Signaling Pathway. Biomed. Pharmacother..

[71] Hu Q., Zhang W., Wu Z., Tian X., Xiang J., Li L., Li Z., Peng X., Wei S., Ma X. (2021). Baicalin and the Liver-Gut System: Pharmacological Bases Explaining Its Therapeutic Effects. Pharmacol. Res..

[72] Yang J., Li M., Zhang C.L., Liu D. (2021). Pharmacological Properties of Baicalin on Liver Diseases: A Narrative Review. Pharmacol. Rep..

[73] Ganguly R., Gupta A., Pandey A.K. (2022). Role of Baicalin as a Potential Therapeutic Agent in Hepatobiliary and Gastrointestinal Disorders: A Review. J. Gastroenterol..

[74] Jia R., Du J., Cao L., Feng W., Xu P., Yin G. (2021). Effects of Dietary Baicalin Supplementation on Growth Performance, Antioxidative Status and Protection against Oxidative Stress-Induced Liver Injury in GIFT Tilapia (Oreochromis Niloticus). Comp. Biochem. Physiol. Part C Toxicol. Pharmacol..

[75] Li P., Chen Y., Ke X., Zhang R., Zuo L., Wang M., Chen Z., Luo X., Wang J. (2022). Baicalin Ameliorates Alcohol-Induced Hepatic Steatosis by Suppressing SREBP1c Elicited PNPLA3 Competitive Binding to ATGL. Arch. Biochem. Biophys..

[76] Fang P., Yu M., Shi M., Bo P., Gu X., Zhang Z. (2020). Baicalin and Its Aglycone: A Novel Approach for Treatment of Metabolic Disorders. Pharmacol. Rep..

[77] Baradaran Rahimi V., Askari V.R., Hosseinzadeh H. (2021). Promising Influences of *Scutellaria baicalensis* and Its Two Active Constituents, Baicalin, and Baicalein, against Metabolic Syndrome: A Review. Phytother. Res..

[78] Ahmadi A., Mortazavi Z., Mehri S., Hosseinzadeh H. (2022). Protective and Therapeutic Effects of *Scutellaria baicalensis* and Its Main Active Ingredients Baicalin and Baicalein against Natural Toxicities and Physical Hazards: A Review of Mechanisms. DARU J. Pharm. Sci..

[79] Ahmadi A., Mortazavi Z., Mehri S., Hosseinzadeh H. (2022). *Scutellaria baicalensis* and Its Constituents Baicalin and Baicalein as Antidotes or Protective Agents against Chemical Toxicities: A Comprehensive Review. Naunyn Schmiedebergs Arch. Pharmacol..

[80] Zha A., Cui Z., Qi M., Liao S., Yin J., Tan B., Liao P. (2020). Baicalin-Copper Complex Modulates Gut Microbiota, Inflammatory Responses, and Hormone Secretion in Don-Challenged Piglets. Animals.

[81] Zha A., Tu R., Cui Z., Qi M., Liao S., Wang J., Tan B., Liao P. (2021). Baicalin–Zinc Complex Alleviates Inflammatory Responses and Hormone Profiles by Microbiome in Deoxynivalenol Induced Piglets. Front. Nutr..

[82] Ming J., Zhuoneng L., Guangxun Z. (2018). Protective Role of Flavonoid Baicalin from *Scutellaria baicalensis* in Periodontal Disease Pathogenesis: A Literature Review. Complement. Ther. Med..

[83] Kunimatsu R., Kimura A., Sakata S., Tsuka Y., Yoshimi Y., Abe T., Kado I., Yashima Y., Izumino J., Nakatani A. (2022). Effects of Baicalin on the Proliferation and Expression of OPG and RANKL in Human Cementoblast-Lineage Cells. J. Dent. Sci..

[84] Kimura A., Kunimatsu R., Yoshimi Y., Tsuka Y., Awada T., Horie K., Gunji H., Abe T., Nakajima K., Kitagawa M. (2018). Baicalin Promotes Osteogenic Differentiation of Human Cementoblast Lineage Cells via the Wnt/β Catenin Signaling Pathway. Curr. Pharm. Des..

[85] Kunimatsu R., Kimura A., Tsuka Y., Horie K., Yoshimi Y., Awada T., Gunji H., Abe T., Nakajima K., Sakata S. (2020). Baicalin Inhibits Root Resorption during Tooth Movement in a Rodent Model. Arch. Oral Biol..

[86] Wei Q., Hao X., Lau B.W.-M., Wang S., Li Y. (2022). Baicalin Regulates Stem Cells as a Creative Point in the Treatment of Climacteric Syndrome. Front. Pharmacol..

[87] Zhou R., Han X., Wang J., Sun J. (2015). Baicalin May Have a Therapeutic Effect in Attention Deficit Hyperactivity Disorder. Med. Hypotheses.

[88] Zhu Y., Zhong L., Peng J., Yuan Q., Xu A. (2019). The Therapeutic Effects of Baicalin on Vitiligo Mice. Biol. Pharm. Bull.

[89] Zhang J.A., Luan C., Huang D., Ju M., Chen K., Gu H. (2020). Induction of Autophagy by Baicalin through the AMPK-MTOR Pathway Protects Human Skin Fibroblasts from Ultraviolet B Radiation-Induced Apoptosis. Drug Des. Dev. Ther..

[90] Zheng G., Gan L., Jia L.Y., Zhou D.C., Bi S., Meng Z.Q., Guan G.J., Huang M.M., He X., Zhang C.F. (2021). Screen of Anti-Migraine Active Compounds from Duijinsan by Spectrum-Effect Relationship Analysis and Molecular Docking. J. Ethnopharmacol..

[91] Wang H., Jiang Q., Zhang L. (2022). Baicalin Protects against Renal Interstitial Fibrosis in Mice by Inhibiting the TGF-β/Smad Signalling Pathway. Pharm. Biol..

[92] Lai N., Fu X., Hei G., Song W., Wei R., Zhu X., Guo Q., Zhang Z., Chu C., Xu K. (2022). The Role of Dendritic Cell Subsets in Recurrent Spontaneous Abortion and the Regulatory Effect of Baicalin on It. J. Immunol. Res..

[93] Chen L., Fan B., Gu H., Yang L., Li X. (2022). Effects of Baicalin on Alopecia and the Associated Mechanism. Biomed. Res. Int..

[94] Xu G., Dou J., Zhang L., Guo Q., Zhou C. (2010). Inhibitory effects of baicalein on the influenza virus in vivo is determined by baicalein in the serum. Biol. Pharm. Bull..

[95] Nayak M.K., Agrawal A.S., Bose S., Naskar S., Bhowmick R., Chakrabarti S., Sarkar S., Chawla-Sarkar M. (2014). Antiviral activity of baicalin against influenza virus H1N1-pdm09 is due to modulation of NS1-mediated cellular innate immune responses. J. Antimicrob. Chemother..

[96] Liu L., Dong Y., Shan X., Li L., Xia B., Wang H. (2019). Anti-depressive effectiveness of baicalin in vitro and in vivo. Molecules.

[97] Lin C.C., Shieh D.E. (1996). In vivo hepatoprotective effect of baicalein, baicalin and wogonin from *Scutellaria rivularis*. Phytother. Res..

[98] Zhang H., Yang X., Zhao L., Jiao Y., Liu J., Zhai G. (2016). In vitro and in vivo study of baicalin-loaded mixed micelles for oral delivery. Drug Deliv..

[99] Hu L., Li L., Yan C., Cao Y., Duan X., Sun J. (2023). Baicalin inhibits airway smooth muscle cells proliferation through the RAS signaling pathway in murine asthmatic airway remodeling model. Oxid. Med. Cell. Longev..

[100] Yanxia G., Jingbo L., Zhili H., Na S., Jianhua G., Xiaozhong Z., Panpan S., Wei Y., Kuohai F., Hongquan L. (2023). Baicalin ameliorates high fat diet-induced nonalcoholic fatty liver disease in mice via adenosine monophosphate-activated protein kinase-mediated regulation of SREBP1/Nrf2/NF-κB signaling pathway. Phytother. Res..

[101] Kim E., Ham S., Jung B.K., Park J.W., Kim J., Lee J.H. (2023). Effect of baicalin on wound healing in a mouse model of pressure ulcers. Int. J. Mol. Sci..

[102] Wang Z., Cheng Y.T., Lu Y., Sun G.Q., Pei L. (2023). Baicalin ameliorates corticosterone-induced depression by promoting neurodevelopment of hippocampal via mTOR/GSK3 β pathway. Chin. J. Integr. Med..

[103] Lu S., Li C., Jin X., Zhu L., Shen J., Bai M., Li Y., Xu E. (2023). Baicalin improves the energy levels in the prefrontal cortex of mice exposed to chronic unpredictable mild stress. Heliyon.

[104] Li H., Cong X., Yu W., Jiang Z., Fu K., Cao R., Tian W., Feng Y. (2023). Baicalin inhibits oxidative injures of mouse uterine tissue induced by acute heat stress through activating the Keap1/Nrf2 signalling pathway. Res. Vet. Sci..

[105] Sun X., Pisano M., Xu L., Sun F., Xu J., Zheng W., Liu X., Zhang Y., Sun R., Cui X. (2023). Baicalin regulates autophagy to onterfere with small intestinal acute graft-versus-host disease. Sci. Rep..

